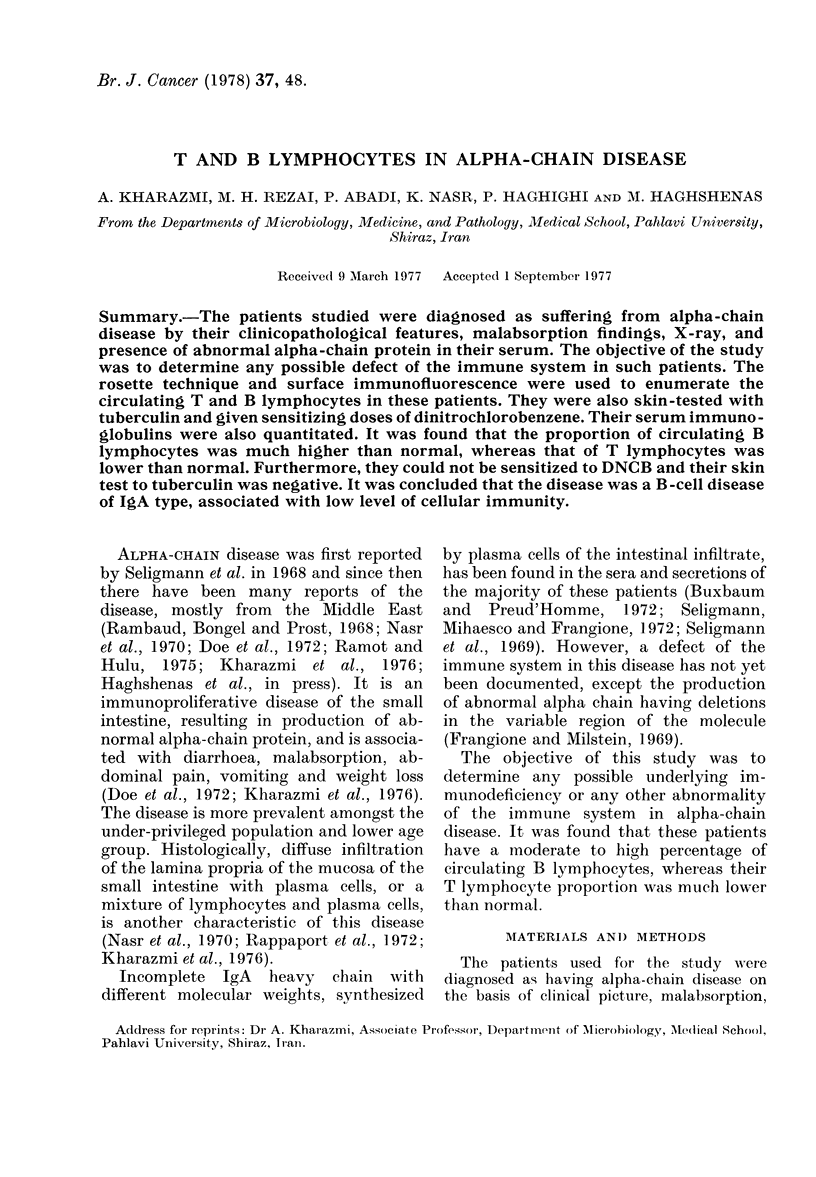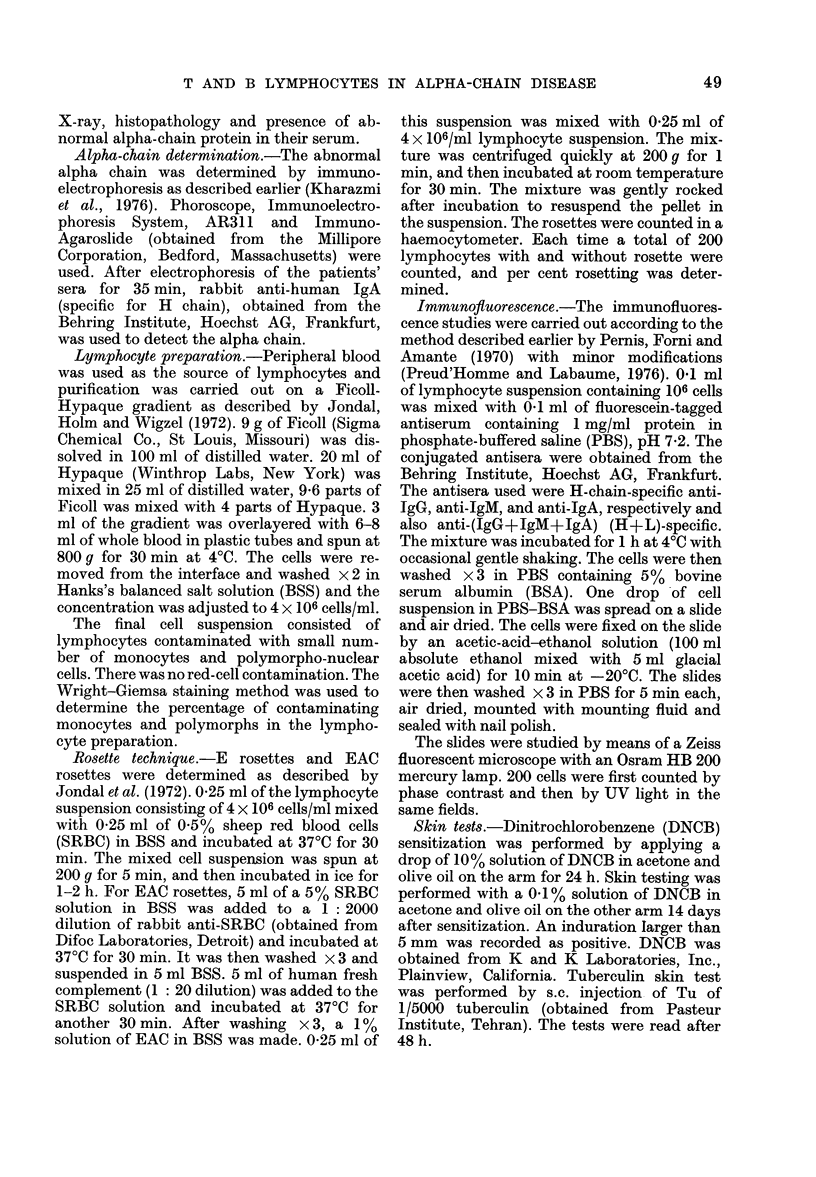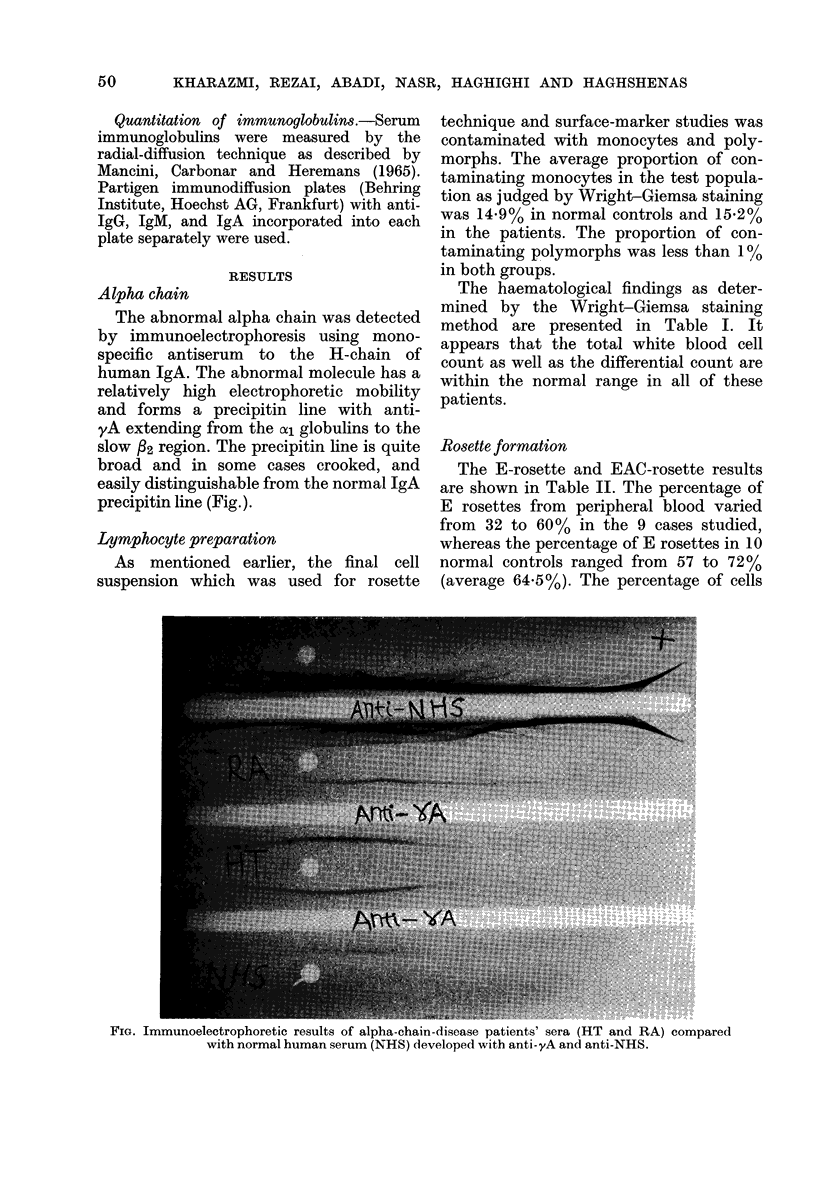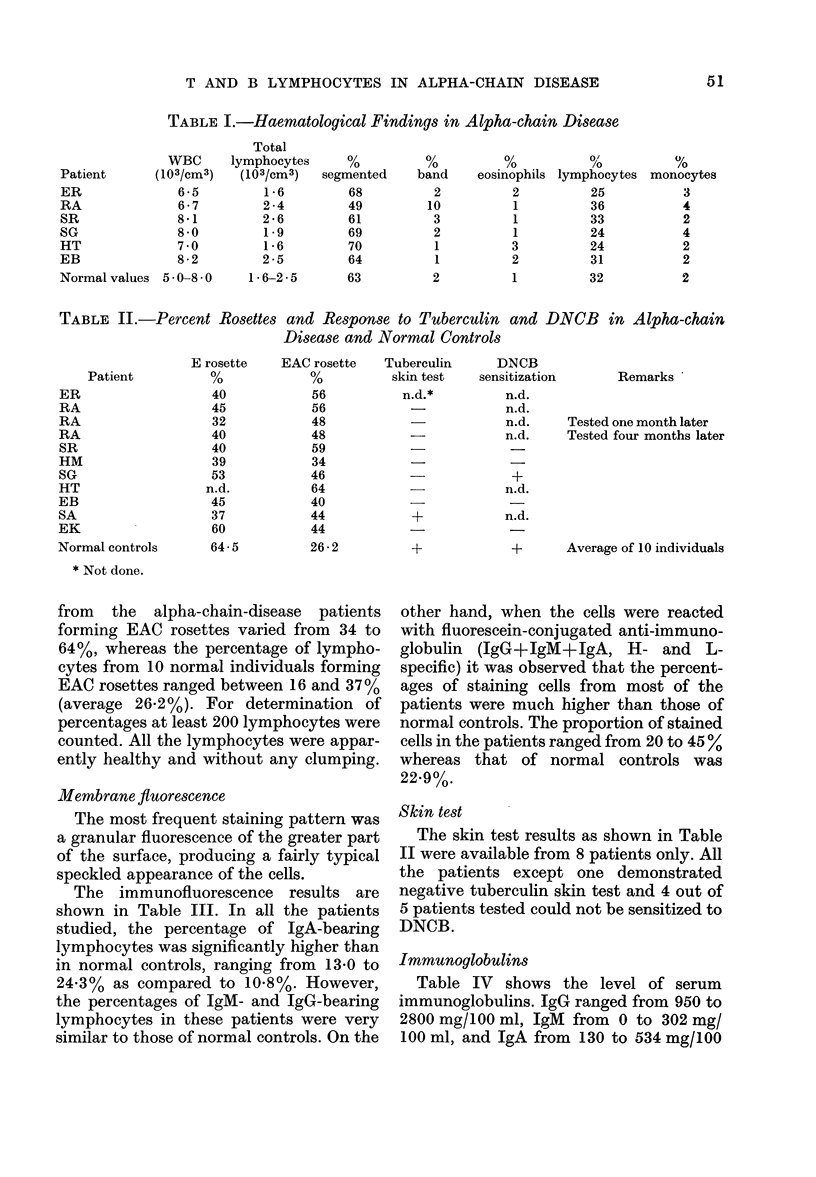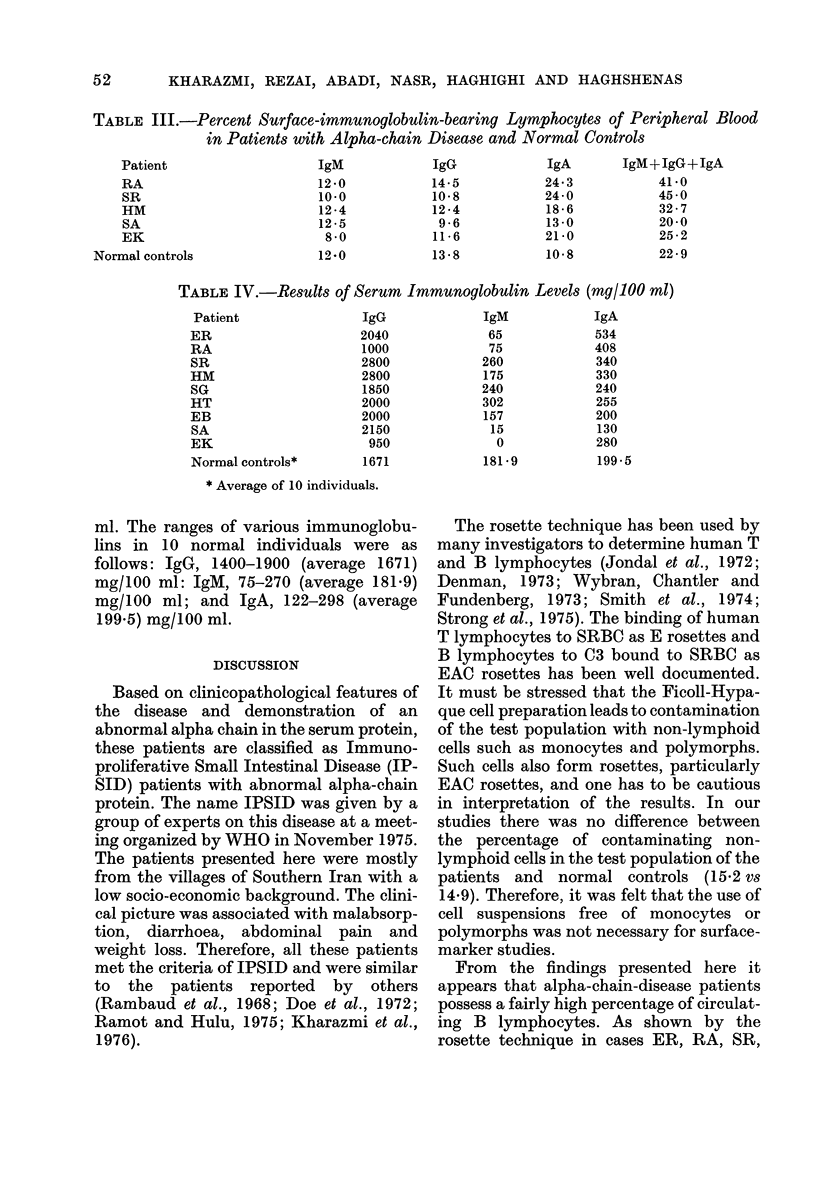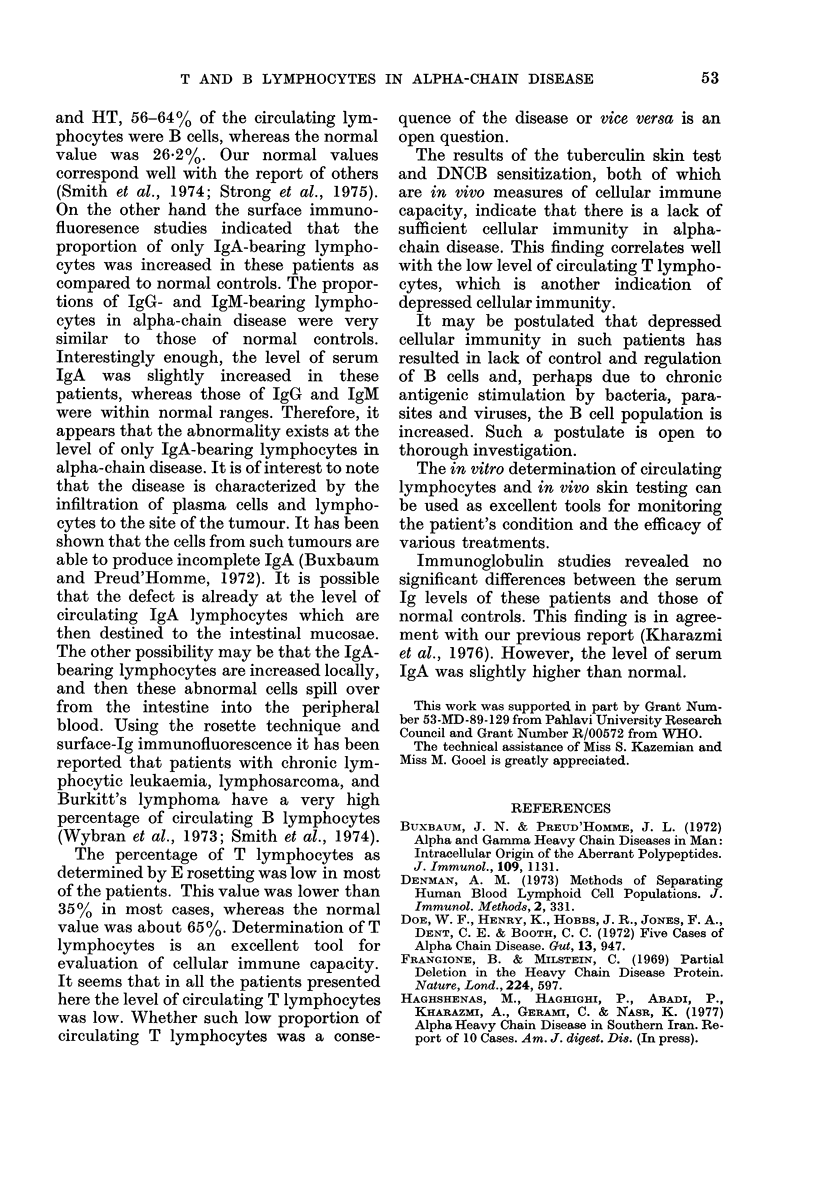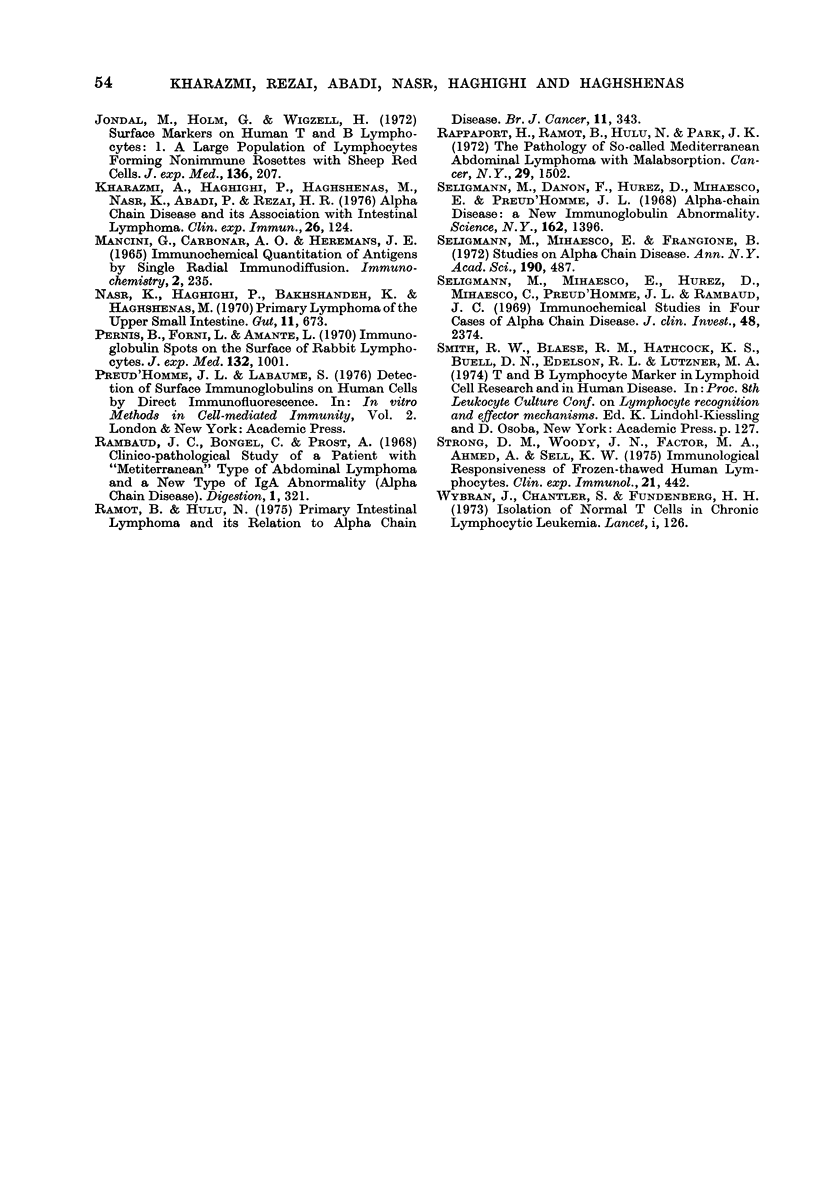# T and B lymphocytes in alpha-chain disease.

**DOI:** 10.1038/bjc.1978.7

**Published:** 1978-01

**Authors:** A. Kharazmi, M. H. Rezai, P. Abadi, K. Nasr, P. Haghighi, M. Haghshenas

## Abstract

**Images:**


*Br. J. Cancer* (1978) **37**, 48.

### T AND B LYMPHOCYTES IN ALPHA-CHAIN DISEASE

A. KHARAZMI, M. H. REZAI, P. ABADI, K. NASR, P. HAGHIGHI AND M. HAGHSHENAS

*From the Departments of Microbiology, Medicine, and Pathology, Medical School, Pahlavi University, Shiraz, Iran*

Received 9 March 1977 Accepted 1 September 1977

**Summary.—The patients studied were diagnosed as suffering from alpha-chain disease by their clinicopathological features, malabsorption findings, X-ray, and presence of abnormal alpha-chain protein in their serum. The objective of the study was to determine any possible defect of the immune system in such patients. The rosette technique and surface immunofluorescence were used to enumerate the circulating T and B lymphocytes in these patients. They were also skin-tested with tuberculin and given sensitizing doses of dinitrochlorobenzene. Their serum immunoglobulins were also quantitated. It was found that the proportion of circulating B lymphocytes was much higher than normal, whereas that of T lymphocytes was lower than normal. Furthermore, they could not be sensitized to DNCB and their skin test to tuberculin was negative. It was concluded that the disease was a B-cell disease of IgA type, associated with low level of cellular immunity.**

ALPHA-CHAIN disease was first reported by Seligmann *et al.* in 1968 and since then there have been many reports of the disease, mostly from the Middle East (Rambaud, Bongel and Prost, 1968; Nasr *et al.*, 1970; Doe *et al.*, 1972; Ramot and Hulu, 1975; Kharazmi *et al.*, 1976; Haghshenas *et al.*, in press). It is an immunoproliferative disease of the small intestine, resulting in production of abnormal alpha-chain protein, and is associated with diarrhoea, malabsorption, abdominal pain, vomiting and weight loss (Doe *et al.*, 1972; Kharazmi *et al.*, 1976). The disease is more prevalent amongst the under-privileged population and lower age group. Histologically, diffuse infiltration of the lamina propria of the mucosa of the small intestine with plasma cells, or a mixture of lymphocytes and plasma cells, is another characteristic of this disease (Nasr *et al.*, 1970; Rappaport *et al.*, 1972; Kharazmi *et al.*, 1976).

Incomplete IgA heavy chain with different molecular weights, synthesized by plasma cells of the intestinal infiltrate, has been found in the sera and secretions of the majority of these patients (Buxbaum and Preud'Homme, 1972; Seligmann, Mihaesco and Frangione, 1972; Seligmann *et al.*, 1969). However, a defect of the immune system in this disease has not yet been documented, except the production of abnormal alpha chain having deletions in the variable region of the molecule (Frangione and Milstein, 1969).

The objective of this study was to determine any possible underlying immunodeficiency or any other abnormality of the immune system in alpha-chain disease. It was found that these patients have a moderate to high percentage of circulating B lymphocytes, whereas their T lymphocyte proportion was much lower than normal.

### MATERIALS AND METHODS

The patients used for the study were diagnosed as having alpha-chain disease on the basis of clinical picture, malabsorption,

Address for reprints: Dr A. Kharazmi, Associate Professor, Department of Microbiology, Medical School, Pahlavi University, Shiraz, Iran.

X-ray, histopathology and presence of abnormal alpha-chain protein in their serum.

*Alpha-chain determination.*—The abnormal alpha chain was determined by immunoelectrophoresis as described earlier (Kharazmi *et al.*, 1976). Phoroscope, Immunoelectrophoresis System, AR311 and Immuno-Agaroslide (obtained from the Millipore Corporation, Bedford, Massachusetts) were used. After electrophoresis of the patients' sera for 35 min, rabbit anti-human IgA (specific for H chain), obtained from the Behring Institute, Hoechst AG, Frankfurt, was used to detect the alpha chain.

*Lymphocyte preparation.*—Peripheral blood was used as the source of lymphocytes and purification was carried out on a Ficoll-Hypaque gradient as described by Jondal, Holm and Wigzel (1972). 9 g of Ficoll (Sigma Chemical Co., St Louis, Missouri) was dissolved in 100 ml of distilled water. 20 ml of Hypaque (Winthrop Labs, New York) was mixed in 25 ml of distilled water, 9·6 parts of Ficoll was mixed with 4 parts of Hypaque. 3 ml of the gradient was overlayered with 6–8 ml of whole blood in plastic tubes and spun at 800 *g* for 30 min at 4°C. The cells were removed from the interface and washed $\times 2$ in Hanks's balanced salt solution (BSS) and the concentration was adjusted to $4 \times 10^6$ cells/ml.

The final cell suspension consisted of lymphocytes contaminated with small number of monocytes and polymorpho-nuclear cells. There was no red-cell contamination. The Wright–Giemsa staining method was used to determine the percentage of contaminating monocytes and polymorphs in the lymphocyte preparation.

*Rosette technique.*—E rosettes and EAC rosettes were determined as described by Jondal *et al.* (1972). 0·25 ml of the lymphocyte suspension consisting of $4 \times 10^6$ cells/ml mixed with 0·25 ml of 0·5% sheep red blood cells (SRBC) in BSS and incubated at 37°C for 30 min. The mixed cell suspension was spun at 200 *g* for 5 min, and then incubated in ice for 1–2 h. For EAC rosettes, 5 ml of a 5% SRBC solution in BSS was added to a 1 : 2000 dilution of rabbit anti-SRBC (obtained from Difoc Laboratories, Detroit) and incubated at 37°C for 30 min. It was then washed $\times 3$ and suspended in 5 ml BSS. 5 ml of human fresh complement (1 : 20 dilution) was added to the SRBC solution and incubated at 37°C for another 30 min. After washing $\times 3$, a 1% solution of EAC in BSS was made. 0·25 ml of this suspension was mixed with 0·25 ml of $4 \times 10^6$/ml lymphocyte suspension. The mixture was centrifuged quickly at 200 *g* for 1 min, and then incubated at room temperature for 30 min. The mixture was gently rocked after incubation to resuspend the pellet in the suspension. The rosettes were counted in a haemocytometer. Each time a total of 200 lymphocytes with and without rosette were counted, and per cent rosetting was determined.

*Immunofluorescence.*—The immunofluorescence studies were carried out according to the method described earlier by Pernis, Forni and Amante (1970) with minor modifications (Preud'Homme and Labaume, 1976). 0·1 ml of lymphocyte suspension containing $10^6$ cells was mixed with 0·1 ml of fluorescein-tagged antiserum containing 1 mg/ml protein in phosphate-buffered saline (PBS), pH 7·2. The conjugated antisera were obtained from the Behring Institute, Hoechst AG, Frankfurt. The antisera used were H-chain-specific anti-IgG, anti-IgM, and anti-IgA, respectively and also anti-(IgG+IgM+IgA) (H+L)-specific. The mixture was incubated for 1 h at 4°C with occasional gentle shaking. The cells were then washed $\times 3$ in PBS containing 5% bovine serum albumin (BSA). One drop of cell suspension in PBS–BSA was spread on a slide and air dried. The cells were fixed on the slide by an acetic-acid–ethanol solution (100 ml absolute ethanol mixed with 5 ml glacial acetic acid) for 10 min at −20°C. The slides were then washed $\times 3$ in PBS for 5 min each, air dried, mounted with mounting fluid and sealed with nail polish.

The slides were studied by means of a Zeiss fluorescent microscope with an Osram HB 200 mercury lamp. 200 cells were first counted by phase contrast and then by UV light in the same fields.

*Skin tests.*—Dinitrochlorobenzene (DNCB) sensitization was performed by applying a drop of 10% solution of DNCB in acetone and olive oil on the arm for 24 h. Skin testing was performed with a 0·1% solution of DNCB in acetone and olive oil on the other arm 14 days after sensitization. An induration larger than 5 mm was recorded as positive. DNCB was obtained from K and K Laboratories, Inc., Plainview, California. Tuberculin skin test was performed by s.c. injection of Tu of 1/5000 tuberculin (obtained from Pasteur Institute, Tehran). The tests were read after 48 h.

*Quantitation of immunoglobulins.*—Serum immunoglobulins were measured by the radial-diffusion technique as described by Mancini, Carbonar and Heremans (1965). Partigen immunodiffusion plates (Behring Institute, Hoechst AG, Frankfurt) with anti-IgG, IgM, and IgA incorporated into each plate separately were used.

### RESULTS

### *Alpha chain*

The abnormal alpha chain was detected by immunoelectrophoresis using monospecific antiserum to the H-chain of human IgA. The abnormal molecule has a relatively high electrophoretic mobility and forms a precipitin line with anti-$\gamma$A extending from the $\alpha_1$ globulins to the slow $\beta_2$ region. The precipitin line is quite broad and in some cases crooked, and easily distinguishable from the normal IgA precipitin line (Fig.).

### *Lymphocyte preparation*

As mentioned earlier, the final cell suspension which was used for rosette technique and surface-marker studies was contaminated with monocytes and polymorphs. The average proportion of contaminating monocytes in the test population as judged by Wright–Giemsa staining was 14·9% in normal controls and 15·2% in the patients. The proportion of contaminating polymorphs was less than 1% in both groups.

The haematological findings as determined by the Wright–Giemsa staining method are presented in Table I. It appears that the total white blood cell count as well as the differential count are within the normal range in all of these patients.

### *Rosette formation*

The E-rosette and EAC-rosette results are shown in Table II. The percentage of E rosettes from peripheral blood varied from 32 to 60% in the 9 cases studied, whereas the percentage of E rosettes in 10 normal controls ranged from 57 to 72% (average 64·5%). The percentage of cells

FIG. Immunoelectrophoretic results of alpha-chain-disease patients' sera (HT and RA) compared with normal human serum (NHS) developed with anti-$\gamma$A and anti-NHS.

TABLE I.—*Haematological Findings in Alpha-chain Disease*

| Patient | WBC ($10^3/cm^3$) | Total lymphocytes ($10^3/cm^3$) | % segmented | % band | % eosinophils | % lymphocytes | % monocytes |
|---|---|---|---|---|---|---|---|
| ER | 6·5 | 1·6 | 68 | 2 | 2 | 25 | 3 |
| RA | 6·7 | 2·4 | 49 | 10 | 1 | 36 | 4 |
| SR | 8·1 | 2·6 | 61 | 3 | 1 | 33 | 2 |
| SG | 8·0 | 1·9 | 69 | 2 | 1 | 24 | 4 |
| HT | 7·0 | 1·6 | 70 | 1 | 3 | 24 | 2 |
| EB | 8·2 | 2·5 | 64 | 1 | 2 | 31 | 2 |
| Normal values | 5·0–8·0 | 1·6–2·5 | 63 | 2 | 1 | 32 | 2 |

TABLE II.—*Percent Rosettes and Response to Tuberculin and DNCB in Alpha-chain Disease and Normal Controls*

| Patient | E rosette % | EAC rosette % | Tuberculin skin test | DNCB sensitization | Remarks |
|---|---|---|---|---|---|
| ER | 40 | 56 | n.d.* | n.d. | |
| RA | 45 | 56 | — | n.d. | |
| RA | 32 | 48 | — | n.d. | Tested one month later |
| RA | 40 | 48 | — | n.d. | Tested four months later |
| SR | 40 | 59 | — | — | |
| HM | 39 | 34 | — | — | |
| SG | 53 | 46 | — | + | |
| HT | n.d. | 64 | — | n.d. | |
| EB | 45 | 40 | — | — | |
| SA | 37 | 44 | + | n.d. | |
| EK | 60 | 44 | — | — | |
| Normal controls | 64·5 | 26·2 | + | + | Average of 10 individuals |

* Not done.

from the alpha-chain-disease patients forming EAC rosettes varied from 34 to 64%, whereas the percentage of lymphocytes from 10 normal individuals forming EAC rosettes ranged between 16 and 37% (average 26·2%). For determination of percentages at least 200 lymphocytes were counted. All the lymphocytes were apparently healthy and without any clumping.

*Membrane fluorescence*

The most frequent staining pattern was a granular fluorescence of the greater part of the surface, producing a fairly typical speckled appearance of the cells.

The immunofluorescence results are shown in Table III. In all the patients studied, the percentage of IgA-bearing lymphocytes was significantly higher than in normal controls, ranging from 13·0 to 24·3% as compared to 10·8%. However, the percentages of IgM- and IgG-bearing lymphocytes in these patients were very similar to those of normal controls. On the other hand, when the cells were reacted with fluorescein-conjugated anti-immunoglobulin (IgG+IgM+IgA, H- and L-specific) it was observed that the percentages of staining cells from most of the patients were much higher than those of normal controls. The proportion of stained cells in the patients ranged from 20 to 45% whereas that of normal controls was 22·9%.

*Skin test*

The skin test results as shown in Table II were available from 8 patients only. All the patients except one demonstrated negative tuberculin skin test and 4 out of 5 patients tested could not be sensitized to DNCB.

*Immunoglobulins*

Table IV shows the level of serum immunoglobulins. IgG ranged from 950 to 2800 mg/100 ml, IgM from 0 to 302 mg/100 ml, and IgA from 130 to 534 mg/100

TABLE III.—*Percent Surface-immunoglobulin-bearing Lymphocytes of Peripheral Blood in Patients with Alpha-chain Disease and Normal Controls*

| Patient | IgM | IgG | IgA | IgM+IgG+IgA |
|---|---|---|---|---|
| RA | 12·0 | 14·5 | 24·3 | 41·0 |
| SR | 10·0 | 10·8 | 24·0 | 45·0 |
| HM | 12·4 | 12·4 | 18·6 | 32·7 |
| SA | 12·5 | 9·6 | 13·0 | 20·0 |
| EK | 8·0 | 11·6 | 21·0 | 25·2 |
| Normal controls | 12·0 | 13·8 | 10·8 | 22·9 |

TABLE IV.—*Results of Serum Immunoglobulin Levels (mg/100 ml)*

| Patient | IgG | IgM | IgA |
|---|---|---|---|
| ER | 2040 | 65 | 534 |
| RA | 1000 | 75 | 408 |
| SR | 2800 | 260 | 340 |
| HM | 2800 | 175 | 330 |
| SG | 1850 | 240 | 240 |
| HT | 2000 | 302 | 255 |
| EB | 2000 | 157 | 200 |
| SA | 2150 | 15 | 130 |
| EK | 950 | 0 | 280 |
| Normal controls* | 1671 | 181·9 | 199·5 |

* Average of 10 individuals.

ml. The ranges of various immunoglobulins in 10 normal individuals were as follows: IgG, 1400–1900 (average 1671) mg/100 ml: IgM, 75–270 (average 181·9) mg/100 ml; and IgA, 122–298 (average 199·5) mg/100 ml.

### DISCUSSION

Based on clinicopathological features of the disease and demonstration of an abnormal alpha chain in the serum protein, these patients are classified as Immunoproliferative Small Intestinal Disease (IPSID) patients with abnormal alpha-chain protein. The name IPSID was given by a group of experts on this disease at a meeting organized by WHO in November 1975. The patients presented here were mostly from the villages of Southern Iran with a low socio-economic background. The clinical picture was associated with malabsorption, diarrhoea, abdominal pain and weight loss. Therefore, all these patients met the criteria of IPSID and were similar to the patients reported by others (Rambaud *et al.*, 1968; Doe *et al.*, 1972; Ramot and Hulu, 1975; Kharazmi *et al.*, 1976).

The rosette technique has been used by many investigators to determine human T and B lymphocytes (Jondal *et al.*, 1972; Denman, 1973; Wybran, Chantler and Fundenberg, 1973; Smith *et al.*, 1974; Strong *et al.*, 1975). The binding of human T lymphocytes to SRBC as E rosettes and B lymphocytes to C3 bound to SRBC as EAC rosettes has been well documented. It must be stressed that the Ficoll-Hypaque cell preparation leads to contamination of the test population with non-lymphoid cells such as monocytes and polymorphs. Such cells also form rosettes, particularly EAC rosettes, and one has to be cautious in interpretation of the results. In our studies there was no difference between the percentage of contaminating non-lymphoid cells in the test population of the patients and normal controls (15·2 *vs* 14·9). Therefore, it was felt that the use of cell suspensions free of monocytes or polymorphs was not necessary for surface-marker studies.

From the findings presented here it appears that alpha-chain-disease patients possess a fairly high percentage of circulating B lymphocytes. As shown by the rosette technique in cases ER, RA, SR, and HT, 56–64% of the circulating lymphocytes were B cells, whereas the normal value was 26·2%. Our normal values correspond well with the report of others (Smith *et al.*, 1974; Strong *et al.*, 1975). On the other hand the surface immunofluoresence studies indicated that the proportion of only IgA-bearing lymphocytes was increased in these patients as compared to normal controls. The proportions of IgG- and IgM-bearing lymphocytes in alpha-chain disease were very similar to those of normal controls. Interestingly enough, the level of serum IgA was slightly increased in these patients, whereas those of IgG and IgM were within normal ranges. Therefore, it appears that the abnormality exists at the level of only IgA-bearing lymphocytes in alpha-chain disease. It is of interest to note that the disease is characterized by the infiltration of plasma cells and lymphocytes to the site of the tumour. It has been shown that the cells from such tumours are able to produce incomplete IgA (Buxbaum and Preud'Homme, 1972). It is possible that the defect is already at the level of circulating IgA lymphocytes which are then destined to the intestinal mucosae. The other possibility may be that the IgA-bearing lymphocytes are increased locally, and then these abnormal cells spill over from the intestine into the peripheral blood. Using the rosette technique and surface-Ig immunofluorescence it has been reported that patients with chronic lymphocytic leukaemia, lymphosarcoma, and Burkitt's lymphoma have a very high percentage of circulating B lymphocytes (Wybran *et al.*, 1973; Smith *et al.*, 1974).

The percentage of T lymphocytes as determined by E rosetting was low in most of the patients. This value was lower than 35% in most cases, whereas the normal value was about 65%. Determination of T lymphocytes is an excellent tool for evaluation of cellular immune capacity. It seems that in all the patients presented here the level of circulating T lymphocytes was low. Whether such low proportion of circulating T lymphocytes was a consequence of the disease or *vice versa* is an open question.

The results of the tuberculin skin test and DNCB sensitization, both of which are *in vivo* measures of cellular immune capacity, indicate that there is a lack of sufficient cellular immunity in alpha-chain disease. This finding correlates well with the low level of circulating T lymphocytes, which is another indication of depressed cellular immunity.

It may be postulated that depressed cellular immunity in such patients has resulted in lack of control and regulation of B cells and, perhaps due to chronic antigenic stimulation by bacteria, parasites and viruses, the B cell population is increased. Such a postulate is open to thorough investigation.

The *in vitro* determination of circulating lymphocytes and *in vivo* skin testing can be used as excellent tools for monitoring the patient's condition and the efficacy of various treatments.

Immunoglobulin studies revealed no significant differences between the serum Ig levels of these patients and those of normal controls. This finding is in agreement with our previous report (Kharazmi *et al.*, 1976). However, the level of serum IgA was slightly higher than normal.

This work was supported in part by Grant Number 53-MD-89-129 from Pahlavi University Research Council and Grant Number R/00572 from WHO.

The technical assistance of Miss S. Kazemian and Miss M. Gooel is greatly appreciated.

### REFERENCES

Buxbaum, J. N. & Preud'Homme, J. L. (1972) Alpha and Gamma Heavy Chain Diseases in Man: Intracellular Origin of the Aberrant Polypeptides. *J. Immunol.*, **109**, 1131.

Denman, A. M. (1973) Methods of Separating Human Blood Lymphoid Cell Populations. *J. Immunol. Methods*, **2**, 331.

Doe, W. F., Henry, K., Hobbs, J. R., Jones, F. A., Dent, C. E. & Booth, C. C. (1972) Five Cases of Alpha Chain Disease. *Gut*, **13**, 947.

Frangione, B. & Milstein, C. (1969) Partial Deletion in the Heavy Chain Disease Protein. *Nature, Lond.*, **224**, 597.

Haghshenas, M., Haghighi, P., Abadi, P., Kharazmi, A., Gerami, C. & Nasr, K. (1977) Alpha Heavy Chain Disease in Southern Iran. Report of 10 Cases. *Am. J. digest. Dis.* (In press).

JONDAL, M., HOLM, G. & WIGZELL, H. (1972) Surface Markers on Human T and B Lymphocytes: 1. A Large Population of Lymphocytes Forming Nonimmune Rosettes with Sheep Red Cells. *J. exp. Med.*, **136**, 207.

KHARAZMI, A., HAGHIGHI, P., HAGHSHENAS, M., NASR, K., ABADI, P. & REZAI, H. R. (1976) Alpha Chain Disease and its Association with Intestinal Lymphoma. *Clin. exp. Immun.*, **26**, 124.

MANCINI, G., CARBONAR, A. O. & HEREMANS, J. E. (1965) Immunochemical Quantitation of Antigens by Single Radial Immunodiffusion. *Immunochemistry*, **2**, 235.

NASR, K., HAGHIGHI, P., BAKHSHANDEH, K. & HAGHSHENAS, M. (1970) Primary Lymphoma of the Upper Small Intestine. *Gut*, **11**, 673.

PERNIS, B., FORNI, L. & AMANTE, L. (1970) Immunoglobulin Spots on the Surface of Rabbit Lymphocytes. *J. exp. Med.* **132**, 1001.

PREUD'HOMME, J. L. & LABAUME, S. (1976) Detection of Surface Immunoglobulins on Human Cells by Direct Immunofluorescence. In: *In vitro Methods in Cell-mediated Immunity*, Vol. 2. London & New York: Academic Press.

RAMBAUD, J. C., BONGEL, C. & PROST, A. (1968) Clinico-pathological Study of a Patient with "Metiterranean" Type of Abdominal Lymphoma and a New Type of IgA Abnormality (Alpha Chain Disease). *Digestion*, **1**, 321.

RAMOT, B. & HULU, N. (1975) Primary Intestinal Lymphoma and its Relation to Alpha Chain Disease. *Br. J. Cancer*, **11**, 343.

RAPPAPORT, H., RAMOT, B., HULU, N. & PARK, J. K. (1972) The Pathology of So-called Mediterranean Abdominal Lymphoma with Malabsorption. *Cancer, N.Y.*, **29**, 1502.

SELIGMANN, M., DANON, F., HUREZ, D., MIHAESCO, E. & PREUD'HOMME, J. L. (1968) Alpha-chain Disease: a New Immunoglobulin Abnormality. *Science, N.Y.*, **162**, 1396.

SELIGMANN, M., MIHAESCO, E. & FRANGIONE, B. (1972) Studies on Alpha Chain Disease. *Ann. N.Y. Acad. Sci.*, **190**, 487.

SELIGMANN, M., MIHAESCO, E., HUREZ, D., MIHAESCO, C., PREUD'HOMME, J. L. & RAMBAUD, J. C. (1969) Immunochemical Studies in Four Cases of Alpha Chain Disease. *J. clin. Invest.*, **48**, 2374.

SMITH, R. W., BLAESE, R. M., HATHCOCK, K. S., BUELL, D. N., EDELSON, R. L. & LUTZNER, M. A. (1974) T and B Lymphocyte Marker in Lymphoid Cell Research and in Human Disease. In: *Proc. 8th Leukocyte Culture Conf.* on *Lymphocyte recognition and effector mechanisms*. Ed. K. Lindohl-Kiessling and D. Osoba, New York: Academic Press. p. 127.

STRONG, D. M., WOODY, J. N., FACTOR, M. A., AHMED, A. & SELL, K. W. (1975) Immunological Responsiveness of Frozen-thawed Human Lymphocytes. *Clin. exp. Immunol.*, **21**, 442.

WYBRAN, J., CHANTLER, S. & FUNDENBERG, H. H. (1973) Isolation of Normal T Cells in Chronic Lymphocytic Leukemia. *Lancet*, i, 126.